# Coronary Microvascular Dysfunction: Epidemiology, Pathophysiology, Diagnosis, and Treatment

**DOI:** 10.1002/mco2.70875

**Published:** 2026-07-22

**Authors:** Zhao Ge, MaoZe Zhang, Xujin Ning, Yijia Du, Lishuo Su, Xudong Wu, Jingyi Zhang, Tongzuo Liu, Xianliang Wang

**Affiliations:** ^1^ First Teaching Hospital of Tianjin University of Traditional Chinese Medicine Tianjin China; ^2^ National Clinical Research Center for Chinese Medicine Tianjin China

**Keywords:** coronary microvascular dysfunction, diagnosis, NRG‐1/ErbB signaling pathway, traditional Chinese medicine, treatment

## Abstract

Coronary microvascular dysfunction (CMD) is a key contributor to myocardial ischemia and cardiovascular diseases. It is characterized by abnormalities of the coronary microvasculature, leading to impaired myocardial perfusion. Although CMD has a high prevalence in patients with nonobstructive coronary artery disease and is associated with adverse cardiovascular events, its pathogenesis has not yet been fully elucidated. Current diagnostic approaches combine noninvasive and invasive methods, but there remains a lack of effective targeted therapies. This review discusses the epidemiology, pathophysiology, diagnostic strategies, and treatment options for CMD, with a particular focus on the protective role of the neuregulin‐1 (NRG‐1)/v‐erb‐b2 erythroblastic leukemia viral oncogene homolog B (ErbB) signaling pathway. We initially outline how the NRG‐1/ErbB pathway affects endothelial function, ventricular remodeling, oxidative stress, and myocardial angiogenesis, highlighting its potential as a therapeutic target. In addition, we explore emerging evidence that traditional Chinese medicine (TCM) interventions may regulate the NRG‐1/ErbB axis to improve microvascular function and cardiac outcomes. Overall, this review deepens our understanding of the mechanisms underlying CMD and provides new avenues for integrated precision therapies, including TCM, with the aim of improving clinical management and prognosis in patients with CMD.

## Graphical abstract

This visual summary illustrates the key aspects of coronary microvascular dysfunction (CMD) in angina with nonobstructive coronary artery disease (NOCAD), including epidemiology, pathophysiology (microvascular remodeling and endothelial dysfunction), the protective NRG‐1/ErbB signaling pathway, diagnostic methods, and both Western and traditional Chinese treatment strategies.

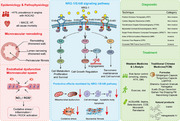

## 1 Introduction

Coronary microvascular dysfunction (CMD) refers to a group of clinical syndromes characterized by structural and functional abnormalities of the coronary microvasculature, resulting in insufficient coronary blood flow to meet myocardial oxygen demand and consequently leading to myocardial ischemia [1, 2, 3]. CMD plays a crucial role in the pathogenesis of myocardial ischemia [4]. Unlike traditional coronary artery disease (CAD), coronary angiography in CMD patients often does not show significant stenosis, making the diagnosis particularly challenging [5, 6]. Recent studies indicate that 47%–60% of patients presenting with typical angina‐like symptoms but without significant coronary artery lesions on angiography exhibit CMD, which significantly impairs their quality of life and prognosis [7]. Related studies have shown that the prevalence of CMD is approximately 41% among angina patients with nonobstructive CAD [8]. More importantly, CMD is closely associated with an increased risk of major adverse cardiovascular events, significantly increasing the risks of heart failure and all‐cause mortality [9, 10]. Currently, there is a lack of effective treatments and diagnostic biomarkers for CMD [11, 12]. A deeper exploration of its pathological mechanisms will not only improve our understanding of the disease but may also expand treatment options in cardiology.

**FIGURE 1:**
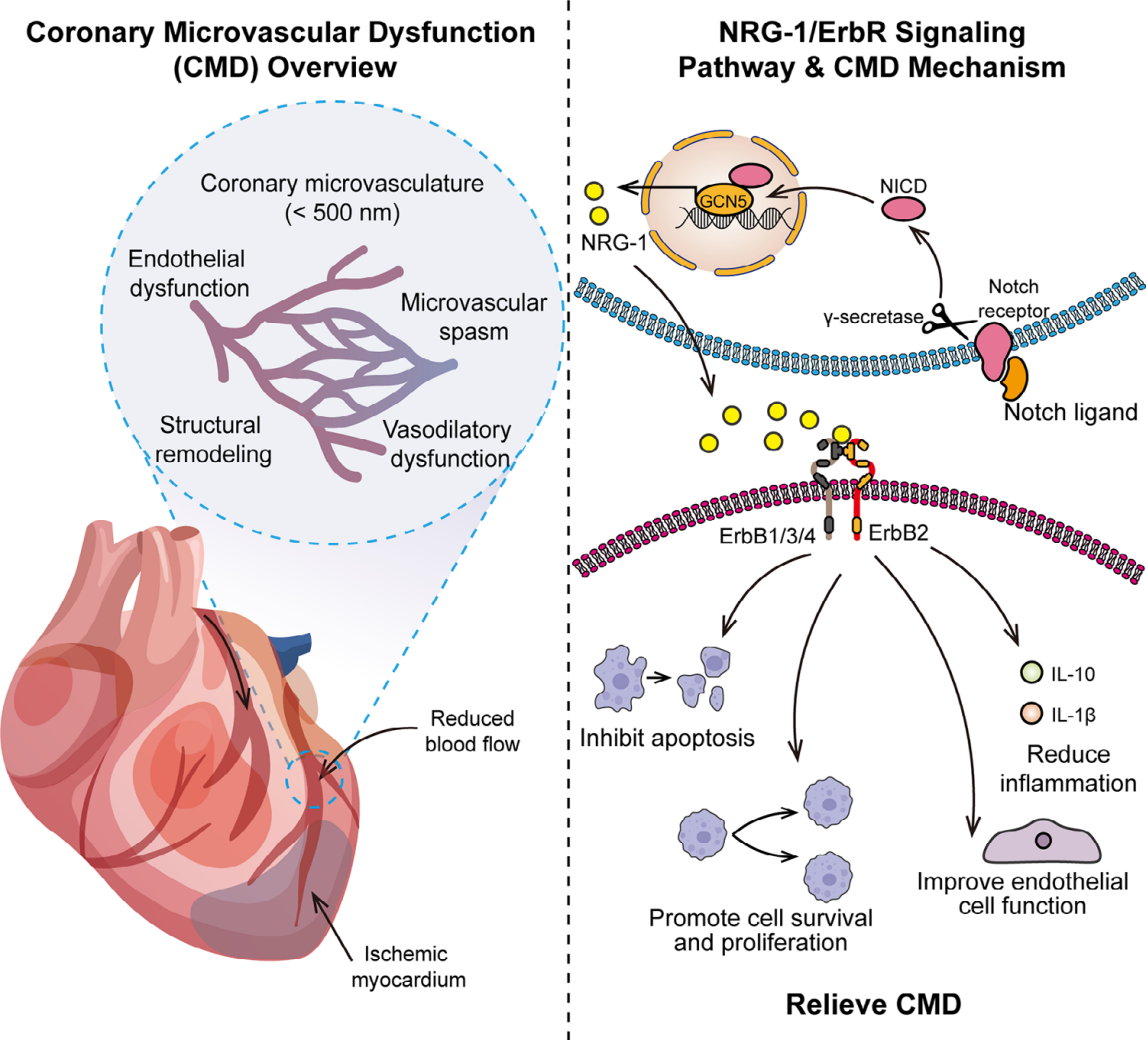
Overview of key pathological features of CMD and potential therapeutic mechanisms targeting the NRG‐1/ErbB pathway. CMD is primarily characterized by endothelial dysfunction, microvascular structural remodeling, and impaired vasodilation. At the molecular level, NRG‐1 activates ErbB receptors (ErbB1/3/4 and ErbB2) and the Notch signaling pathway, and may interact with the histone acetyltransferase GCN5, thereby upregulating anti‐inflammatory factors (such as IL‐10) and inhibiting pro‐inflammatory factors (such as IL‐1β). These effects suppress cell apoptosis, alleviate inflammatory responses, improve endothelial function, and promote cell survival and proliferation, ultimately alleviating CMD.

The pathological mechanisms underlying CMD are complex and include both structural and functional alterations [13, 14, 15]. Structural changes include microvascular remodeling, vascular wall infiltration, luminal obstruction, capillary sparseness, and perivascular fibrosis; functional changes include endothelium‐dependent vasodilation impairment, non‐endothelium‐dependent vasodilation dysfunction, and microvascular spasm [16, 17]. At the molecular level, the occurrence and development of CMD are mainly associated with the excessive production and accumulation of intracellular reactive oxygen species (ROS) and the subsequent oxidative stress response [18, 19]. The accumulation of ROS not only directly impairs the bioavailability of nitric oxide (NO), thereby impairing endothelium‐dependent relaxation, but also activates proapoptotic signals, exacerbating vascular endothelial cell damage and apoptosis, thereby disrupting the integrity of microvascular structures [20, 21]. Given the central role of endothelial dysfunction in the pathogenesis of CMD, targeting molecules that protect endothelial function has become an area of increasing interest [15, 22, 23]. Neuregulin‐1 (NRG‐1) has attracted increasing attention due to its protective effects on vascular endothelial function and cell survival [24, 25]. The Notch signaling pathway, which regulates endothelial function, has been shown to regulate NRG‐1 levels by interacting with the histone acetyltransferase GCN5 [26]. Activation of the Notch signaling pathway enhances NRG‐1 expression, thereby alleviating CMD by improving endothelial function and reducing apoptosis in vascular endothelial cells [26]. This suggests that targeting NRG‐1 could be a promising strategy to mitigate CMD and its associated complications (Figure 1).

NRG‐1 plays a critical regulatory role in the cardiovascular system by activating a family of receptor erythroblastic oncogene B (ErbB) tyrosine kinase receptors [24]. ErbB belongs to the epidermal growth factor (EGF) receptor family, consisting of extracellular, transmembrane, and cytoplasmic tyrosine kinase domains [27]. Four subtypes have been identified in this family to date: ErbB1, ErbB2, ErbB3, and ErbB4. NRG‐1 acts as a ligand for ErbB3 and ErbB4 [28, 29]. The binding of NRG‐1 to ErbB3 or ErbB4 receptors induces the formation of homodimers and heterodimers with each other or with ErbB2 [30], which is vital in maintaining structural and functional integrity in the physiological and pathological states of the heart [31, 32]. Recent studies have shown that activation of the NRG‐1/ErbB signaling pathway inhibits cardiomyocyte apoptosis, promotes cell proliferation, alleviates inflammation, and inhibits myocardial fibrosis [29, 33]. In a rat model of Type 1 diabetes mellitus combined with myocardial infarction, exogenous administration of NRG‐1β improved cardiac function and reversed ventricular remodeling (VR) by inhibiting myocardial fibrosis, apoptosis, and oxidative stress, suggesting that the NRG‐1/ErbB signaling pathway exerts cardioprotective effects under this pathological condition [34]. The NRG‐1/ErbB signaling system also plays an important role in maintaining cardiac function and reversing myocardial remodeling in models of ischemic cardiomyopathy characterized by myocardial fibrosis [35]. Thus, the NRG‐1/ErbB signaling pathway is crucial in cardiomyocytes and is closely related to the normal function of cardiomyocytes and cell injury following myocardial infarction.

It is worth noting that traditional Chinese medicine (TCM) has demonstrated unique application value in the treatment of CMD [36, 37]. TCM has accumulated rich experience in improving the clinical symptoms of CMD patients through syndrome differentiation and individualized treatment. Modern research shows that many Chinese herbal formulas and their active ingredients can protect vascular endothelial function, improve microcirculatory perfusion, and promote myocardial angiogenesis by regulating the NRG‐1/ErbB signaling pathway and its downstream networks [38, 39, 40, 41]. This article aims to systematically review the epidemiological characteristics, pathophysiological mechanisms, diagnostic assessment strategies, and treatment progress of CMD, with a focus on the role of the NRG‐1/ErbB signaling pathway in its development and progression. It explores innovative TCM applications based on this pathway for the treatment of CMD. The article first outlines the epidemiological characteristics and disease burden of CMD, then elucidates its pathological mechanisms at the molecular, functional, and structural levels, with a focus on the role of the NRG‐1/ErbB pathway in regulating vascular endothelial function, VR, and myocardial angiogenesis. On this basis, it systematically introduces noninvasive and invasive diagnostic methods for CMD, reviews existing treatment strategies and emerging therapies, and summarizes the research progress of TCM in treating CMD through the NRG‐1/ErbB pathway. Finally, it provides an outlook on future research directions in the field of CMD, aiming to serve as a systematic reference for basic research and clinical diagnosis and treatment of CMD.

## 2 Epidemiology

The true prevalence of CMD in cardiovascular disease remains unclear, but recent epidemiological studies have provided important data. Existing research indicates that CMD has a higher prevalence in individuals with symptoms of myocardial ischemia but without obstructive CAD [42, 43], especially in patients with angina pectoris associated with nonobstructive CAD [14, 44, 45, 46]. A large systematic review and meta‐analysis showed that the pooled prevalence of CMD in angina patients with nonobstructive CAD was approximately 41%, and it was more common in female patients [8]. Looking at the overall population undergoing coronary angiography, CMD also has a high potential prevalence. Studies showed that among patients who undergo coronary angiography due to clinical symptoms, up to 49% were found to have no obvious coronary artery stenosis; however, among these patients, the proportion who may have CMD can reach 60% [47]. In addition, Kong et al. included 103 patients suspected of having myocardial ischemia in their study, and the results showed that 79% of the patients had CMD, of which 88% were nonobstructive CAD patients and 8% were obstructive CAD patients [48]. This study further demonstrates that CMD is not only widespread in patients with nonobstructive CAD but can also coexist in patients with obstructive CAD and may jointly participate in the occurrence and development of myocardial ischemia. These data suggest that microcirculatory disturbance is a common pathological basis in patients with ischemia that cannot be explained by conventional coronary angiography [48].

In addition to its high prevalence, CMD also has significant prognostic implications [49]. Previously, patients with chest pain and normal coronary angiography or only nonobstructive lesions were considered to have a better overall prognosis. However, increasing research indicates that these patients are not truly low risk. Compared to individuals without ischemic heart disease, patients with angina but normal coronary arteries or nonobstructive lesions have a significantly increased risk of major adverse cardiovascular events, with a 52% increased risk in those with normal coronary arteries and an 85% increased risk in those with diffuse nonobstructive lesions [50]. This result indicates that even without significant epicardial coronary artery stenosis, patients may still be at higher risk of cardiovascular events due to microcirculatory abnormalities. Therefore, CMD should no longer be regarded as a simple functional abnormality or benign clinical condition, but should be incorporated into long‐term cardiovascular risk assessment for ischemic heart disease. In addition, CMD not only significantly increases the risk of heart failure and all‐cause mortality [9, 10], but is also closely associated with recurrent angina attacks, increased hospitalization rates, and decreased quality of life [50]. Therefore, clarifying the epidemiological characteristics of CMD is crucial for identifying high‐risk populations, optimizing prevention strategies, and guiding subsequent clinical management.

## 3 Pathophysiological Mechanisms

In recent years, as research has progressed, our understanding of the pathological mechanisms of CMD has evolved from a single‐level description to a systematic understanding of the synergistic effects of multiple dimensions and mechanisms. The occurrence and development of CMD are not determined by a single isolated factor, but result from the combined, mutually reinforcing effects of multiple mechanisms, including anatomical structural abnormalities, molecular regulatory disorders, function–environment interactions, and neuroendocrine imbalances [51, 52, 53]. These mechanisms exhibit complex hierarchical relationships: structural abnormalities can directly lead to hemodynamic changes, thereby exacerbating oxidative stress and inflammatory responses at the molecular level; while molecular disorders further damage vascular structure and function, forming a vicious cycle. The complex interrelationships among these mechanisms collectively determine the clinical phenotypic heterogeneity of CMD and provide multidimensional potential intervention targets for its diagnosis and treatment.

### 3.1 Structural Changes

**FIGURE 2:**
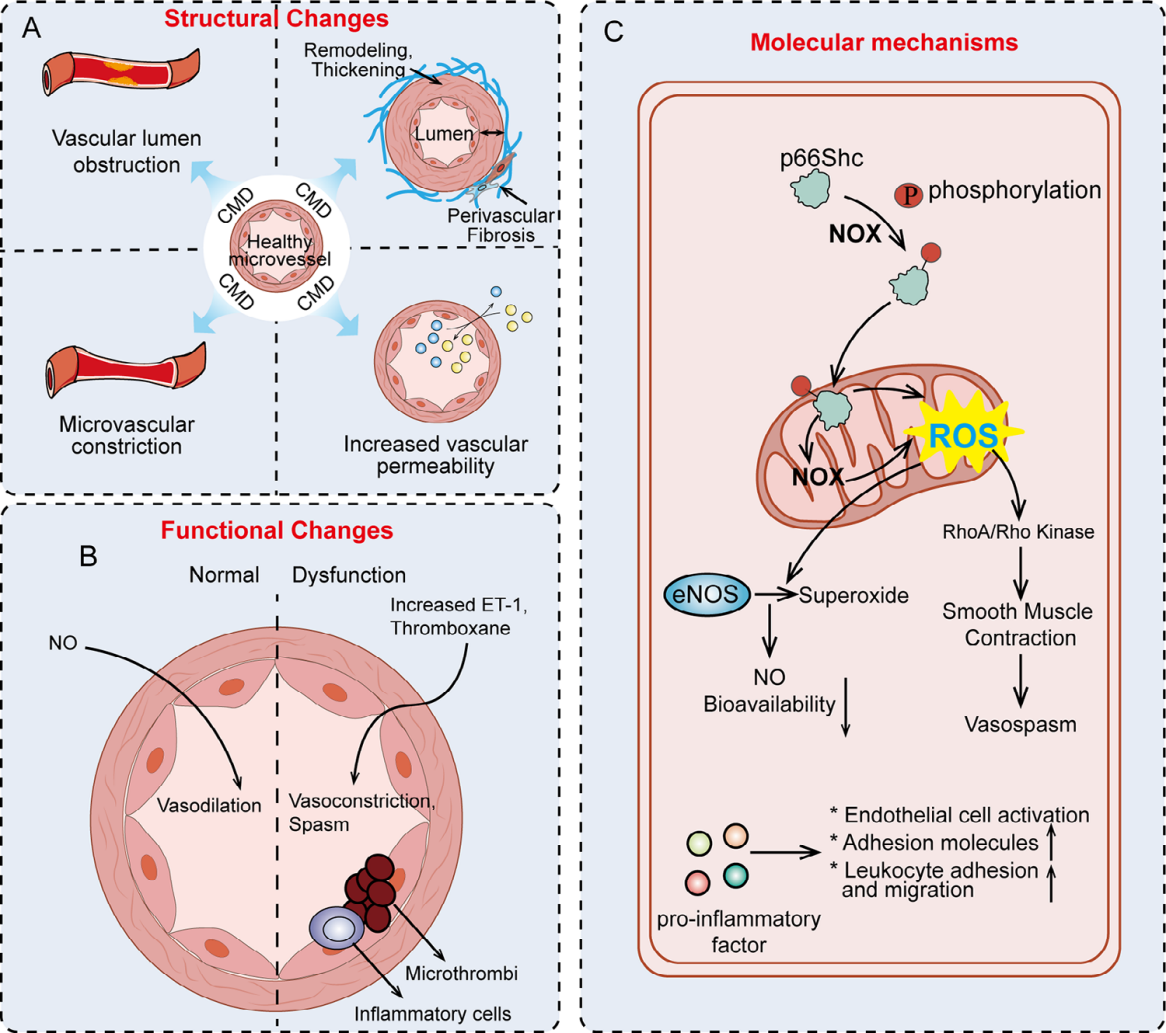
Core pathological mechanisms of CMD. (A) Structural changes: Compared with healthy microvessels, CMD microvessels exhibit medial thickening, vascular wall remodeling, luminal narrowing, and capillary rarefaction, leading to increased microcirculatory resistance. (B) Functional changes: Endothelial dysfunction results in reduced vasodilatory factors (e.g., NO) and increased vasoconstrictors (e.g., ET‐1, thromboxane), triggering vasoconstriction and spasm, along with inflammatory cell infiltration and microthrombus formation, further exacerbating microcirculatory perfusion impairment. (C) Molecular mechanisms: Oxidative stress (ROS) creates a vicious cycle through the Nox and p66Shc pathways, leading to eNOS uncoupling, reduced NO bioavailability, and activation of the RhoA/Rho kinase pathway, which promotes smooth muscle contraction, vascular spasm, and inflammatory responses, collectively driving the onset and progression of CMD.

The triggers of CMD include structural abnormalities and functional abnormalities of the coronary microvasculature. Coronary microvascular structural abnormalities include microvascular constriction, microvascular remodeling, vessel wall infiltration, lumen obstruction, vessel thinning, and perivascular fibrosis [54, 55] (Figure 2). Microvascular constriction is commonly observed in hypertrophic cardiomyopathy and hypertension. It is characterized by thickening of the media of the interventricular arteriole, often accompanied by intimal thickening, resulting in a slight reduction in the arteriole's lumen area [56]. Studies have shown that in patients with obesity, smoking, diabetes, and other risk factors, as well as atherosclerosis and other diseases, coronary microvessels can exhibit centripetal remodeling, which is manifested by thickening of the vessel wall, narrowing of the lumen, and decreased myocardial capillary density [57]. Coronary microvascular remodeling increases microcirculation resistance and reduces perfusion volume. Its response to non‐endothelium‐dependent vasodilators is characterized by decreased coronary flow reserve (CFR) and increased coronary index of microvascular resistance (IMR) [58]. A decreased CFR reflects impaired maximal vasodilation capacity of microvessels, while an elevated IMR directly quantifies increased microcirculatory resistance. These abnormalities in functional parameters are important indicators of CMD severity and guide clinical intervention.

### 3.2 Functional Changes

Abnormal function of coronary microvessels includes smooth muscle dysfunction, endothelial dysfunction, and autonomic nervous system dysfunction [59]. Functional abnormalities may result from impaired dilation of the precoronary arterioles and arterioles or increased microvascular constriction caused by damage to the vascular endothelium or the release of vasoactive substances.

Endothelial injury and dysfunction are among the important mechanisms of CMD. Most cardiovascular risk factors can impair endothelium‐dependent vasodilation by reducing the production and bioavailability of NO, or by decreasing the production and increasing the degradation of vasodilators such as prostacyclin and endothelial hyperpolarizing factor, thereby causing an imbalance in microvascular vasomotor regulation and increasing the tendency for microvascular spasm [60, 61, 62]. Furthermore, in acute ischemia‐reperfusion‐related microcirculatory injury, swollen endothelial cells can directly cause capillary stenosis or even occlusion, and the large amount of vasoactive substances released during reperfusion can further aggravate microvascular vasodilatory dysfunction [63].

Simultaneously, endothelial injury can also activate platelets and promote fibrin deposition through oxidative stress and abnormal activation of vasoactive substances, leading to microthrombus formation. This can manifest in different clinical contexts, such as risk factor‐related in situ microthrombus formation, or as epicardial plaque fragment detachment or interventional procedure‐related microembolism. These pathological processes increase microcirculatory resistance and further exacerbate microcirculatory perfusion impairment via microthrombosis, inflammation, and chronic vascular wall remodeling [64, 65]. Furthermore, autonomic nervous system dysfunction also contributes to the development of CMD; excessive sympathetic nerve activation can enhance microvascular vasoconstriction and may mutually reinforce endothelial dysfunction, forming a progressively worsening pathological loop [66] (Figure 2).

### 3.3 Molecular Mechanisms

Oxidative stress resulting from the excessive intracellular production and accumulation of reactive ROS, together with subsequent inflammatory responses, is recognized as a key pathogenic mechanism driving CMD progression [18] (Figure 2). Nicotinamide adenine dinucleotide phosphate ester oxidase (Nox) subtypes and mitochondria represented the major systems regulating ROS production [67]. Nox activation leads to ROS generation, triggering p66Shc phosphorylation and mitochondrial translocation. In mammals, p66Shc is a proapoptotic protein that further enhances ROS production [18]. In turn, p66Shc activation stimulates Nox activity, creating a vicious cycle of increased ROS. Increased intracellular ROS concentration promotes the conversion of NO to peroxynitrite and leads to the uncoupling of endothelial nitric oxide synthase (eNOS) [68]. After eNOS transforms from an enzyme that produces NO to one that produces superoxide, it activates the RhoA/Rho kinase pathway, a member of the small G protein Ras homolog family, leading to impaired NO‐mediated vasodilation and enhanced ET‐1‐mediated vasoconstrictive effect [69, 70, 71]. The RhoA/Rho kinase pathway was closely associated with ROS production and excessive contraction of vascular smooth muscle cells (VSMCs), which regulate smooth muscle contractility through calcium sensitivity and the phosphorylation of contractile muscle filaments. Therefore, it is thought to be responsible for the susceptibility of coronary vessels to spasticity and for enhancing inflammation by inducing pro‐inflammatory molecules in VSMCs and endothelial cells [71]. Notably, recent studies have shown that the NRG‐1/ErbB signaling pathway can affect vascular function by regulating the RhoA/ROCK pathway. In a rat sepsis model, NRG‐1 treatment significantly inhibited RhoA and ROCK1 protein expression, reduced vascular permeability, alleviated endothelial damage, and improved cardiac function [29]. These studies suggest that NRG‐1 not only participates in maintaining vascular endothelial barrier function but may also improve microcirculatory blood perfusion by inhibiting RhoA activation, thereby reducing excessive vascular smooth muscle contraction and inflammatory response. The interaction between the NRG‐1/ErbB signaling axis and the RhoA/ROCK pathway may jointly participate in the occurrence and development of CMD, providing theoretical and experimental evidence for further exploration of NRG‐1 intervention in this pathway to treat CMD.

Inflammation plays a central role in the pathogenesis of CMD [72]. Pro‐inflammatory mediators such as tumor necrosis factor‐α, interleukin (IL)‐6, and IL‐1β are elevated in CMD patients, adversely affecting the microvascular system [73]. Studies by Kopeva et al. showed that CMD patients have significantly higher levels of inflammatory biomarkers than non‐CMD patients [74]. Chronic inflammation impairs microvascular structure and function by inducing endothelial cell activation, increasing adhesion molecule expression, and promoting leukocyte adhesion and migration [75].

The elucidation of the aforementioned molecular mechanisms reveals the central role of oxidative stress, inflammatory responses, and the RhoA/ROCK signaling pathway in the pathogenesis of CMD. Furthermore, recent studies have found that the NRG‐1/ErbB signaling pathway, as an important protective signaling axis in the cardiovascular system, plays a crucial role in maintaining vascular endothelial function, reducing myocardial damage, and promoting vascular repair by regulating the RhoA/ROCK pathway and inhibiting oxidative stress and inflammatory responses. This pathway not only interacts closely with the aforementioned molecular mechanisms but may also serve as a potential hub connecting multiple pathological aspects of CMD.

### 3.4 The Role of the NRG‐1/ErbB Signaling Pathway in CMD

#### 3.4.1 Overview of NRG‐1/ErbB Signaling Pathway

**FIGURE 3:**
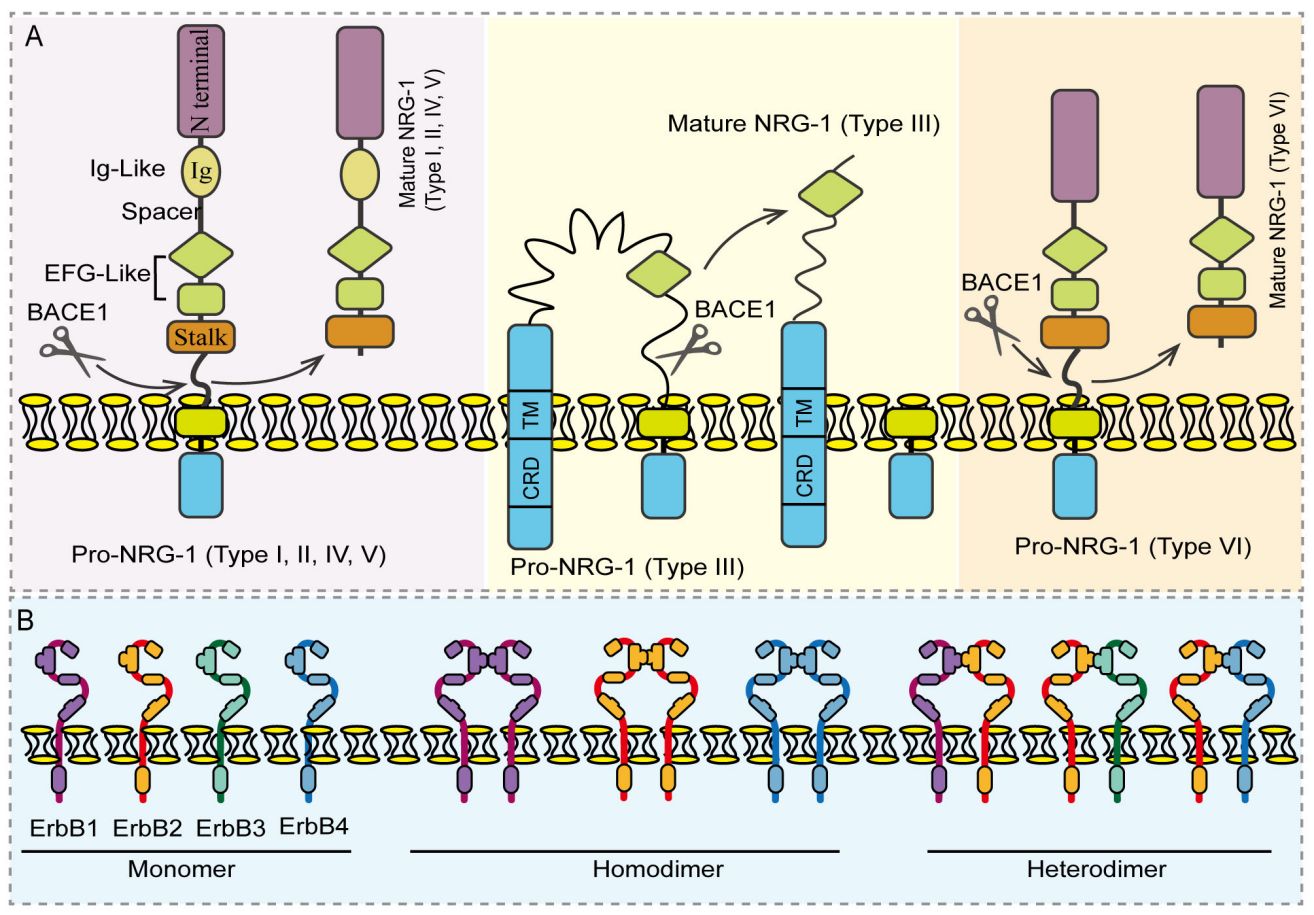
Processing and maturation of NRG‐1 and its initiation of signal transduction through the ErbB receptor family. (A) Precursors of different NRG‐1 isoforms (Pro‐NRG‐1) are cleaved by BACE1 to generate mature NRG‐1, which harbors key functional modules including an Ig‐like domain, a spacer region, and an EGF‐like domain. (B) Upon binding to ErbB receptors, mature NRG‐1 induces the formation of homodimers or heterodimers. ErbB4 possesses a complete kinase domain and can form functional homodimers; ErbB3 lacks a kinase domain and cannot form homodimers; ErbB2 primarily serves as a co‐receptor involved in heterodimer assembly.

NRG represents a group of growth factors encoded by four distinct genes (*NRG* 1–4), all of which contain an EGF‐like domain [76] (Figure 3). NRG‐1 is one of the most highly regarded proteins in this family and can be classified into six types and more than 30 isoforms based on differences in the amino acid terminal regions [77]. The EGF structural domain allows NRG‐1 to act as a ligand for the ErbB [78]. NRG‐1 performs its biological roles by triggering the ErbB family. The ErbB family includes the receptors ErbB1, ErbB2, ErbB3, and ErbB4, which share considerable structural similarities [27]. ErbB4 is distinct due to its ligand‐binding and kinase domains, which allow it to form functional homodimers or heterodimers upon NRG‐1 binding [79]. On the other hand, ErbB3 cannot form homologous dimers in dimer complexes or phosphorylate other ErbB receptors because it lacks functional kinase domains [80]. At the same time, ErbB2 homologous dimers are catalytically inactive, and ErbB2 acts as a co‐receptor primarily by forming heterodimers with other ErbB receptors [81]. NRG‐1β binds ErbB4, forming a homodimer or heterodimer with one of the other ErbBs, leading to activation and signal transduction of intracellular tyrosine kinase domains and recruiting adaptor/effector molecules such as p85, Src, and Shc (subunits of PI3K), thus conveying signals to their transducers.

**FIGURE 4:**
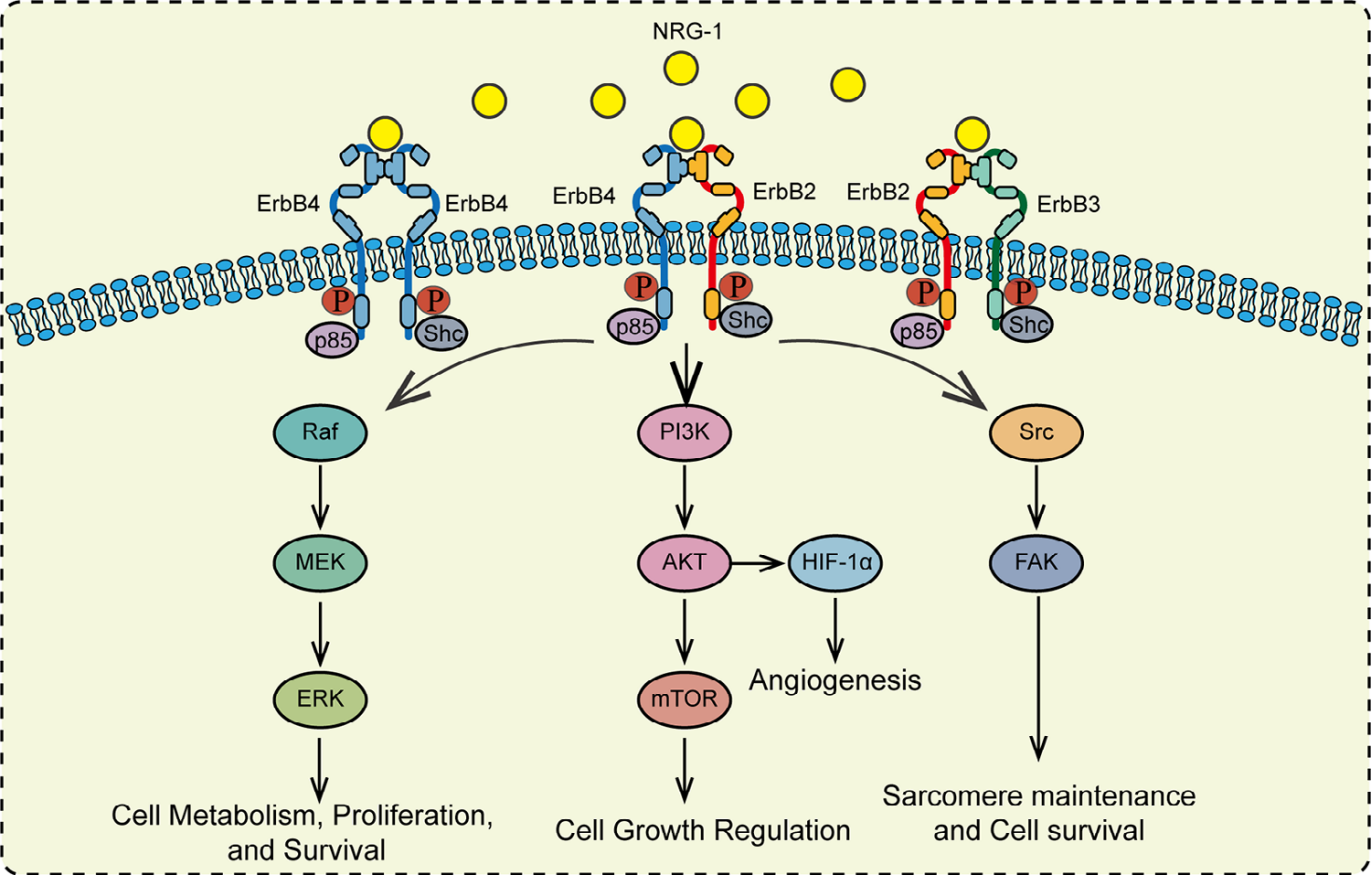
Schematic diagram of the mechanisms by which NRG‐1 activates the Raf‐MEK‐ERK, PI3K‐Akt‐mTOR, and Src/FAK signaling pathways through ErbB receptors. Upon binding to ErbB family receptors, NRG‐1 initiates three major downstream signaling pathways through homodimerization or heterodimerization. NRG‐1 binds to ErbB4 to form homodimers, or forms heterodimers with ErbB2 and ErbB3, thereby activating the Raf‐MEK‐ERK, PI3K‐Akt‐mTOR, and Src/FAK pathways, respectively. The Raf‐MEK‐ERK pathway is primarily involved in regulating cell metabolism, proliferation, and survival. The PI3K‐Akt‐mTOR pathway, mediated by ErbB2 heterodimerization or ErbB4 homodimerization, activates downstream HIF‐1α, promoting angiogenesis, cell metabolism, and survival. The Src/FAK pathway participates in cell survival and sarcomere maintenance through the formation of focal adhesion complexes. Together, these three pathways coordinate the biological functions of NRG‐1 in angiogenesis, cardiomyocyte protection, and cell growth regulation.

NRG‐1‐induced ErbB receptor homologous or heterodimer activation typically initiates the Raf‐MEK‐ERK, PI3K‐Akt‐mTOR, and Src/FAK pathways [82, 83] (Figure 4). As a major downstream effector of ErbB, Raf‐MEK‐ERK regulates cell metabolism, proliferation, and survival [84]. Studies have shown that the Raf‐MEK‐ERK pathway plays an important role in various cell types, particularly in vascular endothelial cells, where its activation is closely linked to cellular metabolic activity. Specifically, TGF‐β1 induces glutaminolysis in endothelial cells through the PP2A‐regulated Raf‐MEK‐ERK signaling pathway, a process critical for cell survival under hypoxic and inflammatory conditions [85]. Furthermore, the Raf‐MEK‐ERK signaling pathway also plays a key role in the proliferation and survival of vascular endothelial cells. Research indicates that vascular endothelial growth factor (VEGF) promotes endothelial cell proliferation and migration by activating VEGFR2 and the Raf/MEK/ERK cascade [86]. Activation of this pathway not only promotes cell proliferation but also enhances cell survival by regulating the expression of cell cycle‐related genes [87]. After nerve injury, NRG‐1 activates the MEK/ERK pathway through ErbB receptors, promoting microglial cell proliferation and survival, further demonstrating the importance of this pathway in cell survival [88].

The PI3K‐Akt‐mTOR pathway is another pathway activated by NRG‐1. Upon binding to ErbB3 or ErbB4, NRG‐1 promotes heterodimerization of these receptors with ErbB2. On the one hand, ErbB receptors undergo transphosphorylation, which activates Akt [89]. On the other hand, ErbB4 or ErbB3 can directly bind PI3K, and upregulation of ErbB receptors activates the ErbB/PI3K/Akt signaling pathway [90]. NRG‐1‐induced PI3K‐dependent Akt activation in cardiomyocytes is closely linked to cell survival, metabolism, and growth regulation [34, 91, 92]. The mechanism by which NRG‐1 modulates Akt signaling to protect cardiomyocytes remains partially unclear. Overexpression of NRG‐1 in endothelial progenitor cells (EPCs) enhances its autocrine levels and upregulates p‐PI3K and p‐Akt expression, thereby reducing infarct size in myocardial infarction mice and promoting myocardial vascular regeneration [93]. NRG‐1 activation of the PI3K/Akt/mTOR pathway also leads to HIF‐1α activation and regulation of angiogenesis [94]. Sequence analyses indicate that ErbB4 contains a consensus motif homologous to the PI3K‐binding sequence in ErbB3. While ErbB3 is regarded as the principal PI3K‐docking subunit among ErbB receptors, ErbB4 can also engage PI3K in a subtype‐dependent manner. Accordingly, NRG‐1‐induced PI3K/Akt activation does not necessarily require ErbB3 under conditions where ErbB4 is activated, potentially including ErbB4 homodimers at sufficiently high ligand concentrations [95, 96].

The third pathway of NRG‐1 activation involved focal adhesion kinase (FAK). FAK is a Src‐binding protein. FAK recruits and activates Src to form a complex that jointly regulates cell growth and survival [97], a process crucial for vascular regeneration [98]. In cardiomyocytes, NRG‐1/ErbB activates FAK to maintain sarcomere and cell survival [99]. Adult rat ventricular myocytes are treated with cimaglermin alfa (GGF2) to induce the formation of multiprotein complexes containing ErbB2, FAK, p130CAS, and paxillin, which induce stress fiber formation and myocyte elongation. Finally, they restored the contact and synchronous beating between myocytes [100]. Recombinant NRG‐1 induces Src‐dependent phosphorylation of FAK at tyrosine residue 861 in myocytes to form focal adhesion complexes [100]. Pretreatment with a specific antibody against ErbB2 can effectively block NRG‐1‐induced FAK phosphorylation and the formation of focal adhesion complexes [101]. These results suggest that NRG‐1/ErbB might regulate FAK to normalize sarcomere load by coordinating myocardial cell growth and division.

#### 3.4.2 Regulation of Vascular Endothelial Function by NRG‐1/ErbB Signaling Pathway

**FIGURE 5:**
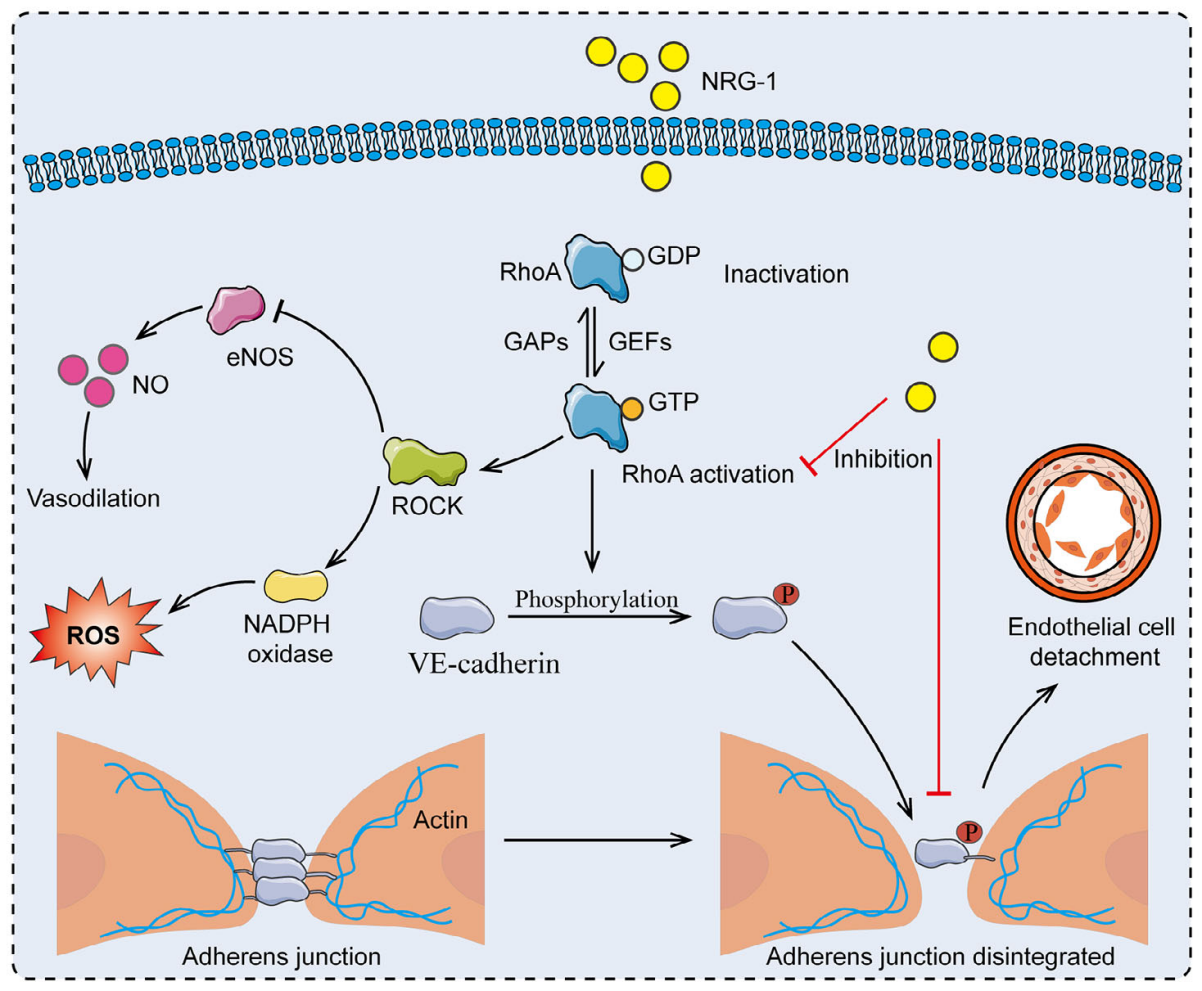
Schematic diagram of the mechanism by which NRG‐1 regulates endothelial cell function through the RhoA/ROCK signaling pathway. RhoA is converted into its active GTP‐bound form by guanine nucleotide exchange factors (GEFs), or inactivated into its GDP‐bound form by GTPase‐activating proteins (GAPs). Activated RhoA mediates multiple downstream effects through ROCK: it inhibits eNOS activity, reducing NO production and leading to impaired vasodilation; activates NADPH oxidase, promoting ROS production and exacerbating oxidative stress; and induces VE‐cadherin phosphorylation, disrupting adherens junction integrity and increasing endothelial barrier permeability. NRG‐1 can inhibit RhoA activation, thereby maintaining F‐actin cytoskeleton structure and preserving endothelial barrier function, suggesting its potential protective role in ameliorating vascular dysfunction.

Vascular endothelial dysfunction (VED) plays a significant role in the development of cardiovascular diseases [102]. Because endothelial cells are located at the boundary between circulating blood and tissue, they are most susceptible to various pathogenic factors, such as ROS. The hallmark of VED is impaired endothelium‐dependent vasodilation. A healthy endothelium regulates vascular tone by producing vasodilatory factors such as NO and prostacyclin (PGI2) [103]. RhoA, a prominent member of the Rho guanosine triphosphate (GTPase) family and that is extensively researched, plays a crucial role in actin polymerization and cell migration [104]. RhoA is activated by guanine nucleotide exchange factors (GEFs) to form GTP‐bound forms and can also be inactivated by GTPase‐activating proteins (GAPs) to form GDP‐bound forms [105]. Studies have shown that activation of the RhoA/ROCK signaling pathway can inhibit eNOS activity, thereby reducing NO production and impairing vasodilation [106]. The RhoA/ROCK pathway is involved in oxidative stress, leading to the activation of NADPH oxidase and subsequent ROS production in cells [107]. In addition, activated RhoA can phosphorylate VE‐cadherin at Y731, leading to structural instability of the VE‐cadherin‐β‐catenin complex, which opens adherens junctions, disrupts cell–cell contact, and impairs endothelial barrier function [108, 109]. Notably, Wu et al. confirmed that NRG‐1 could inhibit IL‐1β‐induced RhoA activation, thereby reducing vascular permeability, maintaining F‐actin cytoskeleton structure, and maintaining endothelial barrier function [110]. However, studies have shown that diabetes induces vascular dysfunction by activating the EGFR/ErbB2‐ROCKpathway [111]. Although this disease model differs significantly from CMD, it also suggests that other potential adverse consequences of NRG‐1‐activated ErbB2 cannot be ignored, and appropriate animal models need to be established for further exploration. The above studies suggest that NRG‐1 enhances endothelial cell secretory function and modulates cardiac endothelial cell permeability, which may be mediated by the RhoA/ROCK signaling pathway (Figure 5).

Platelet endothelial cell adhesion molecule‐1 (PECAM‐1) plays an important role in maintaining endothelial junction stability, regulating transendothelial migration, and angiogenesis [112]. Research indicates that PECAM‐1 functions as a mechanical sensor in endothelial cells, enabling them to detect and respond to hemodynamic forces generated by blood flow [113]. Although PECAM‐1 specifically initiates mechanical signaling in endothelial cells, its presence can profoundly affect interactions between endothelial cells and other cell types, such as VSMCs, thereby impacting the overall function and health of blood vessels. The absence of PECAM‐1 in endothelial cells leads to increased NRG‐1 release and elevated NO/ROS signaling, resulting in abnormal activation of ErbB2 receptors in cardiomyocytes and impaired contractility [114]. This novel PECAM‐1/NRG‐1/ErbB2 pathway could be a crucial mechanism that harmonizes communication between cardiac endothelial cells and cardiomyocytes.

#### 3.4.3 Function of NRG‐1/ErbB Signaling Pathway in VR

The left ventricle and arterial system are anatomically continuous, making their interactions a key determinant of cardiovascular function [114]. CMD is influenced by microvascular remodeling, which is closely associated with VR [115]. VR refers to the changes in the structure and morphology of the heart resulting from various insults [116]. The key feature of pathological VR is the loss of cardiomyocytes due to myocardial fibrosis, apoptosis, and necrosis [117]. Studies have found that intravenous infusion of recombinant isoforms of NRG‐1 can inhibit myocardial fibrosis to prevent adverse remodeling after injury [118]. In the monocrotaline‐induced pulmonary hypertension rat model, recombinant human NRG‐1 (rhNRG‐1, 40 µg/kg/day) can significantly reduce right ventricular dysfunction in the model group, potentially due to activation of the NRG‐1/ErbB pathway [119]. NRG‐1 can also prevent cardiac remodeling by inhibiting oxidative stress. The NRG‐1/ErbB4 system in the heart is activated early in heart failure to enhance cardiomyocyte resistance to oxidative stress [120]. Treatment of coronary artery ligated rats with rhNRG‐1 (10 µg/kg/day) significantly alleviated cardiac dysfunction and left VR in rats, a mechanism that may be related to the reduction of mitochondrial dysfunction, oxidative stress, and apoptosis [121]. Gupte et al. further demonstrated that NRG‐1β treatment of myocardial infarction rats was associated with activation of the ErbB pathway [35]. Preclinical and clinical (Phases II and III) studies have demonstrated that recombinant NRG‐1 therapy improves myocardial contractility and attenuates left VR [120]. This evidence suggests that NRG‐1 can slow down VR by inhibiting cardiac fibrosis, apoptosis, and oxidative stress.

#### 3.4.4 NRG‐1/ErbB Signaling Pathway and Myocardial Angiogenesis and Survival

Myocardial angiogenesis plays a crucial role in myocardial development, growth, and recovery from injury. In the heart, NRG‐1 is produced and secreted by endocardial and cardiac microvascular endothelial cells (CMECs), playing a crucial role in cardiovascular system development and in sustaining adult heart function [122, 123]. ErbB receptors are expressed in endothelial cells, and NRG‐1 regulates their proliferation, function, and angiogenesis. NRG‐1 promotes the proliferation of vascular endothelial cells in vitro, independent of VEGF [124]. Recently, there has been increasing evidence that NRG‐1 is a positive regulator of angiogenesis, endothelium‐produced NRG‐1 is necessary for femoral artery ligation‐induced angiogenesis and arteriogenesis, and exogenous NRG‐1 can further boost this mechanism [125]. NRG‐1 mitigates vascular damage by enhancing the density of myocardial capillaries in diabetic cardiomyopathy rats [126]. NRG‐1 can influence angiogenesis by inhibiting the apoptosis of EPCs [127]. EPCs are progenitor cells derived from bone marrow that can be recruited to active angiogenic sites to promote tissue repair [128]. ErbB receptors are expressed in EPCs and stimulation with NRG‐1 improves survival and regulates angiogenesis in EPCs [127]. The downstream signaling mechanisms underlying NRG‐1‐induced angiogenesis are not fully understood. It is well known that angiogenesis is regulated by VEGF [129]. It has been suggested that NRG‐1 induces VEGF expression in other tissues or cells. For example, NRG‐1 upregulates VEGF expression in periodontal ligament stem cells in a dose‐dependent manner, thereby enhancing tube formation [130]. Addition of exogenous NRG‐1 promotes VEGF expression and increases pro‐angiogenic responses in human brain microvascular endothelial cells and astrocytes exposed to excessive hemoglobin and ischemic conditions, which is related to ErbB4 signal transduction [131]. Interestingly, Wu et al. found that ErbB2 expression was much higher than that of ErbB3 and ErbB4 in human CMECs (HCMECs). Treatment of hypoxia/serum deprivation in HCMECs with VEGF (100 ng/mL) can upregulate the expression and secretion of NRG‐1, suggesting that VEGF may regulate myocardial angiogenesis and survival through the NRG‐1/ErbB pathway [132]. Collectively, these studies highlight the potential of NRG‐1 in facilitating myocardial recovery by boosting angiogenesis. NRG‐1 interaction with ErbB receptors may be a potential mechanism for regulating angiogenesis.

In summary, the NRG‐1/ErbB signaling pathway plays a crucial protective role in the development and progression of CMD by regulating vascular endothelial function, inhibiting oxidative stress and inflammatory responses, improving VR, and promoting myocardial angiogenesis. It is noteworthy that these pathophysiological processes do not exist in isolation but ultimately manifest clinically as impaired microvascular perfusion, endothelial dysfunction, and increased microcirculatory resistance. Therefore, accurate identification of CMD and its different pathological phenotypes requires not only a deeper understanding of its molecular mechanisms but also the use of diagnostic tools that reflect microcirculatory function and myocardial perfusion status. Based on this, the following section will systematically introduce diagnostic techniques for CMD, including noninvasive assessment and invasive functional examination methods, aiming to lay the foundation for subsequent classification, diagnosis, and precision treatment.

## 4 Diagnostic Techniques

The diagnosis of CMD is particularly challenging because the target vessel diameter is < 500 µm, making it impossible to directly visualize using traditional coronary angiography [14, 133]. Therefore, the diagnosis of CMD relies on hemodynamic assessment of microvascular function, requiring a combination of noninvasive and invasive examination methods to form a multimodal assessment system (Table 1) [134].

**TABLE 1:** Main assessment techniques for CMD.

Technique	Category	Core index (threshold)	Main advantage	Main limitation
Positron emission tomography (PET)	Noninvasive	CFR < 2.0	Noninvasive gold standard, high accuracy	Expensive, radiation exposure
Cardiac magnetic resonance (CMR)	Noninvasive	MPRI < 2.0	No radiation, evaluates structure/function/perfusion	Time‐consuming, gadolinium contraindicated
Transthoracic Doppler echocardiography (TTDE)	Noninvasive	CFVR < 2.0	Bedside, noninvasive, low cost, no radiation	Limited to left anterior descending artery
Myocardial contrast echocardiography (MCE)	Noninvasive	Myocardial perfusion reserve	Bedside, no radiation	Operator‐dependent, high variability
Single photon emission computed tomography (SPECT)	Noninvasive	CFR	Good agreement with PET	Lower resolution than PET
Index of microcirculatory resistance (IMR)	Invasive	IMR > 25 U	Good reproducibility, accurate	Requires pressure/temperature guidewire
Coronary flow reserve (CFR)	Invasive	CFR < 2.0	Assesses epicardial + microvascular	Cannot distinguish between the two
Corrected TIMI frame count (CTFC)	Invasive	Corrected frame count	Good reproducibility	Does not directly reflect microvasculature

Noninvasive techniques for coronary microvascular function provide important tools for early screening and functional assessment of CMD. Positron emission tomography (PET) is currently the recognized gold standard for noninvasive assessment of CMD. By quantifying myocardial blood flow (MBF) at rest and during stress, myocardial flow reserve (MFR) is calculated. An MFR < 2.0 suggests the presence of CMD and is closely associated with an increased risk of adverse cardiovascular events [135, 136, 137]. PET technology has high reproducibility [138, 139, 140]. Cardiac magnetic resonance (CMR) imaging can simultaneously assess cardiac structure, function, and myocardial perfusion. The semi‐quantitative myocardial perfusion reserve index (MPRI) has good diagnostic value for CMD in female patients [141]. Furthermore, CMR can differentiate between myocardial ischemia and non‐ischemic causes (such as myocarditis and Takotsubo syndrome) through delayed gadolinium‐enhanced imaging, playing an important role in etiological differentiation in patients with suspected myocardial infarction but unobstructed coronary arteries (MINOCA) [142, 143]. Transthoracic Doppler echocardiography can assess microvascular function by measuring the coronary flow velocity ratio (CFVR), offering advantages including absence of radiation, low cost, and high reproducibility. However, it is highly operation‐dependent and mainly limited to assessing the left anterior descending artery [144, 145].

Invasive assessment of coronary microvascular function is an important method for diagnosing CMD, especially suitable for patients with no obvious occlusion on coronary angiography but still with ischemic symptoms [146, 147, 148]. The core of this approach is to combine vasoactive drug loading with coronary guidewires to directly evaluate endothelium‐independent and endothelium‐dependent microcirculatory function. Endothelium‐independent microvascular dilation is assessed using CFR and the IMR. CFR can be measured using thermodilution or Doppler guidewires, typically with adenosine‐induced maximal congestion; a CFR < 2.0 or 2.5 indicates impaired microvascular dilation [149, 150, 151]. Simultaneously, combining CFR with IMR can further quantify microvascular resistance. IMR ≥ 25 often indicates abnormal microvascular function, and has the advantages of good repeatability and relative independence from the degree of epicardial coronary artery stenosis [150, 152]. For endothelial‐dependent function, the acetylcholine provocation test can be used. If ischemic symptoms and electrocardiogram changes are induced, but there is no obvious epicardial spasm, it suggests microvascular spasm. If significant epicardial contraction occurs, it helps to differentiate angina pectoris complicated by vasospasm [153, 154, 155]. Currently, invasive coronary functional tests can be used to classify and assess CMD, including different phenotypes such as microvascular diastolic dysfunction, microvascular spasm, and combined epicardial spasm, which is of great value for the treatment of CMD [156, 157].

## 5 Treatment Strategies

To date, the optimal treatment for CMD remains undefined, as CMD is not a discrete disease entity but a clinical syndrome driven by multiple mechanisms, including endothelium‐dependent diastolic dysfunction, non‐endothelium‐dependent diastolic dysfunction, microvascular spasm, structural remodeling, and myocardial metabolic abnormalities. Therefore, treatment strategies should emphasize a combination of comprehensive risk factor management, pathophysiological classification‐based guidance, and individualized intervention. In recent years, with the development of coronary functional testing and noninvasive perfusion assessment technologies, CMD treatment is gradually shifting from empirical antianginal therapy to a precision‐based, mechanism‐stratified approach.

### 5.1 Risk Factor Management and Lifestyle Intervention

Risk factor management and lifestyle intervention are the cornerstones of CMD treatment [158]. Traditional cardiovascular risk factors such as smoking, obesity, hypertension, hyperlipidemia, and diabetes are closely related to the occurrence and development of CMD [44, 159, 160, 161]. Related studies have shown that smokers have a 21% lower CFR than normal controls [162]. Similarly, CFR is often reduced in obese individuals without clinical heart disease [163]. Therefore, actively controlling these risk factors is crucial for delaying the progression of microvascular complications. Lifestyle modifications, including physical exercise, smoking cessation, and weight loss, help improve myocardial ischemia symptoms and prognosis in CMD [164, 165], and improve patients' cardiopulmonary exercise tolerance and CFR function. In addition, strict control of blood pressure, blood glucose, and blood lipids is key to maintaining the structural and functional integrity of microvessels [7].

### 5.2 Western Medicine Treatment

Currently, there is a lack of unified, specific, and well‐supported standard treatment regimens for CMD. Existing drugs are mainly selected based on their pathophysiological mechanisms and clinical phenotypes. The core strategy is to improve microvascular dilation, reduce microcirculatory resistance, inhibit microvascular spasm, and alleviate myocardial ischemia. Angiotensin‐converting enzyme inhibitors (ACEIs) and angiotensin II receptor blockers (ARBs) can improve endothelial function by inhibiting the activation of the renin–angiotensin–aldosterone system, reducing oxidative stress and inflammatory responses, and increasing NO bioavailability. Therefore, they have important value in CMD patients with hypertension, diabetes, or a tendency for left VR [166, 167, 168]. Statins, in addition to lowering lipids, also have pleiotropic effects such as stabilizing endothelial function, anti‐inflammation, anti‐oxidation, and improving vasodilation [169, 170]. For CMD patients with dyslipidemia or atherosclerotic risk factors, there is evidence that statins, alone or in combination with other drugs, are beneficial for patients with coronary artery endothelial or vascular smooth muscle dysfunction, including those with nonobstructive CAD [171]. Beta‐blockers are the first‐line treatment for patients with CMD accompanied by exercise‐induced angina [172]. They relieve symptoms by lowering heart rate, prolonging diastole, reducing myocardial oxygen consumption, and improving coronary perfusion. Beta‐blockers often achieve good efficacy in CMD patients with predominantly endothelial dysfunction and decreased perfusion reserve; carvedilol has been shown to improve endothelial function [173, 174]. Calcium channel blockers play an important role in patients with microvascular spasm or vasomotor abnormalities [175, 176]. They have been shown to improve endothelial function and increase the antioxidant capacity of endothelial cells by enhancing NO synthase activity, and are considered the first‐line treatment strategy for epicardial coronary artery spasm [177]. Nicorandil has both nitrate‐like effects and ATP‐sensitive potassium channel opening effects, which can improve coronary microcirculation perfusion and reduce microvascular resistance, and has some potential for controlling angina symptoms in CMD patients [178]. Ranolazine can alleviate symptoms in some patients with refractory angina or metabolic abnormalities by improving myocardial sodium–calcium overload and diastolic function [179].

### 5.3 The Therapeutic Effects of TCM on CMD via Modulation of NRG‐1/ErbB Pathway

**FIGURE 6:**
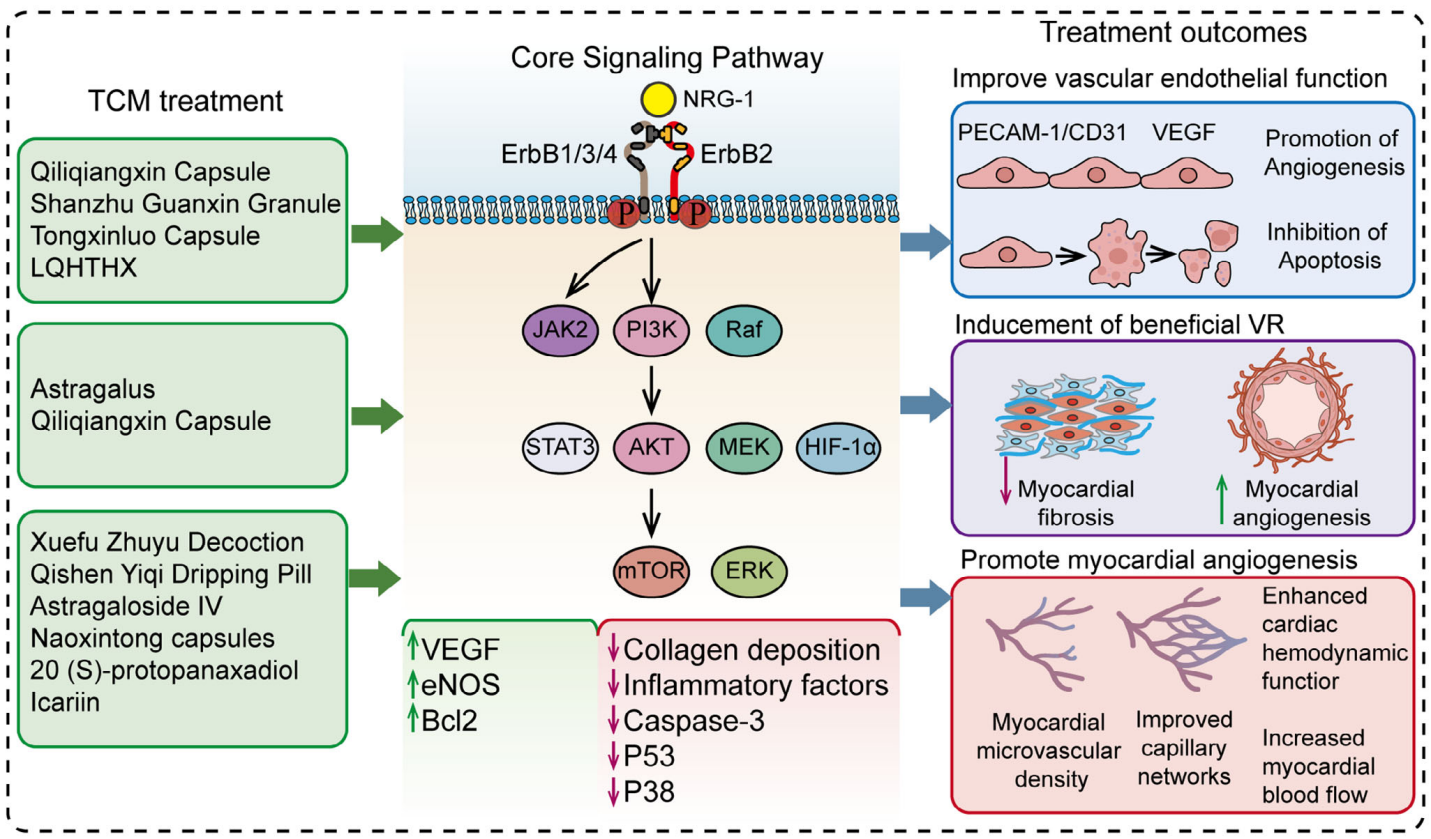
Therapeutic effects of traditional Chinese medicine on CMD based on the NRG‐1/ErbB signaling pathway. Traditional Chinese medicine interventions include Qiliqiangxin capsule, Shenzhu Guanxin granule, Tongxinluo capsule, Liqi Huatan Huoxue (LQHTHX) formula, Astragalus, Xuefu Zhuyu decoction, Qishen Yiqi dripping pill, Naoxintong capsule, 20(S)‐protopanaxadiol, and Icariin. These interventions primarily exert their effects by activating the NRG‐1/ErbB signaling axis, thereby regulating multiple downstream signaling pathways such as PI3K/Akt, Raf/MEK/ERK, and JAK2/STAT3, which mediate a range of biological effects: improving vascular endothelial function (upregulating CD31, VEGF), inducing beneficial ventricular remodeling (inhibiting fibrosis and apoptosis), and promoting myocardial angiogenesis (increasing microvascular density, improving hemodynamics), thus exerting comprehensive therapeutic effects on CMD.

In recent years, due to the significant clinical effects of TCM treatment, more and more studies have focused on TCM treatment of coronary microcirculation disorders [180]. Some extracts or active components of TCM can protect vascular endothelial function, improve VR, promote myocardial angiogenesis, and alleviate coronary microcirculation disorders by regulating the NRG‐1/ErbB signaling pathway (Figure 6). The NRG‐1/ErbB pathway is essential for maintaining myocardial structure and function when cardiomyocytes are subjected to various physiological and pathological stimuli [181].

#### 5.3.1 Improve Vascular Endothelial Function

Vascular endothelial cells play a crucial role in maintaining vascular function, and therefore, preservation of vascular endothelial function is significant for the prevention and treatment of CMD. CMECs are a specific cell type derived from coronary artery microvessels that are rapidly damaged after myocardial ischemia and hypoxia [182]. Qiliqiangxin capsule is a traditional Chinese compound preparation that promotes cardiomyocyte migration, angiogenesis, and reduces apoptosis [183, 184]. The study found that Qiliqiangxin capsule alleviated hypoxic injury in Sprague‐Dawley rat CMECs, characterized by promoting angiogenesis, inhibiting apoptosis, and upregulating the expression of NRG‐1, phosphorylated ErbB2, and phosphorylated ErbB4 [38]. Shenzhu Guanxin granule enhances cardiac hemodynamic function and reduces infarct size by upregulating the expression of PECAM‐1/CD31 and VEGF in a dose‐dependent manner to promote angiogenesis [40]. Tongxinluo (TXL) capsule is a TCM that has been widely used in the treatment of cardiovascular diseases [185, 186]. Previous studies have shown that TXL can improve endothelial function, promote vasodilation, and inhibit inflammatory responses and cell apoptosis [187]. Studies have investigated the ameliorative effect of TXL on myocardial fibrosis after acute myocardial infarction (AMI) and its relationship with vascular endothelial function. The results showed that TXL significantly improved cardiac function, reduced myocardial fibrosis, and inhibited hypoxia‐induced endothelial‐to‐mesenchymal transition (EndMT) in HCMECs, thereby protecting vascular endothelial function [41]. Its mechanism is closely related to the activation of the NRG‐1/ErbB‐PI3K/AKT signaling pathway, and knockdown of NRG‐1 attenuated the protective effect of TXL, suggesting that this pathway plays a critical role in TXL‐mediated improvement of microvascular endothelial function [41]. Furthermore, recent studies have found that active ingredients in LQHTHX (a TCM formula for regulating qi, resolving phlegm, and promoting blood circulation), such as nobiletin, corydaline, and isosinensetin, can enter the bloodstream. In a rat model of CMD induced by sodium urate, LQHTHX exerted protective effects by inhibiting inflammatory responses, reducing collagen deposition, improving mitochondrial function, and inhibiting apoptosis. In hypoxia‐induced CMECs, LQHTHX‐containing serum also reduced cell damage through the same mechanisms. Further mechanistic studies suggest that LQHTHX exerts its therapeutic effect on CMD primarily through a central mechanism centered around the NRG‐1/ErbB‐PI3K/AKT axis [36].

#### 5.3.2 Inducement of Beneficial VR

VR is a key pathological step in the progression of CMD to heart failure, primarily manifested as myocardial fibrosis, cardiomyocyte apoptosis, and ventricular chamber enlargement [188, 189, 190]. The NRG‐1/ErbB signaling pathway plays an important role in inhibiting VR [93, 181]. TCM has shown promising potential in improving VR by regulating this pathway. In a diabetic cardiomyopathy cell model, Astragalus was found to induce NRG‐1 expression, activate ErbB2/4 and the downstream AKT/PI3K signaling pathway, exerting antiapoptotic, anti‐oxidative, and pro‐proliferative effects, thereby inhibiting VR [191]. In addition, Qiliqiangxin capsule has been shown to improve cardiac function and reverse VR in a rat model of heart failure induced by myocardial infarction, as evidenced by reduced heart weight‐to‐body weight ratio and NT‐proBNP levels, alleviated inflammation and fibrosis, promoted myocardial capillary angiogenesis, and inhibited cardiomyocyte apoptosis. The underlying mechanism may involve upregulation of NRG‐1, HIF‐1α, VEGF, and phosphorylated Akt, downregulation of p53 mRNA expression, and modulation of the Bcl‐2/Bax ratio and Caspase‐3 activity, suggesting that Qiliqiangxin capsule exerts pro‐angiogenic and antiapoptotic effects by regulating multiple signaling pathways, including NRG‐1/PI3K/Akt, p53, and HIF‐1α/VEGF [39]. These findings indicate that TCM can intervene in the process of VR through multiple pathways by regulating the NRG‐1/ErbB signaling pathway.

#### 5.3.3 Promote Myocardial Angiogenesis

A reduction in myocardial capillaries is a key pathogenic factor in CMD. Enhancing blood flow capacity, restoring cardiac performance, and optimizing hemodynamics can significantly boost angiogenesis. NRG‐1 upregulates the expression of VEGF, which is closely related to angiogenesis [126, 130, 192]. Xuefu Zhuyu decoction can upregulate the expression of VEGF protein, increase myocardial microvascular density, and promote angiogenesis in acute myocardial ischemia rats [193]. Qishen Yiqi dripping pill promotes myocardial angiogenesis. Clinical studies have shown that preoperative administration of Qishen Yiqi dripping pill in patients undergoing elective percutaneous coronary intervention significantly reduces the postoperative index of microcirculatory resistance and cardiac troponin I levels, suggesting its role in improving coronary microvascular function and alleviating myocardial injury [194]. Furthermore, animal experiments have demonstrated that in a rat model of myocardial infarction, Qishen Yiqi dripping pill significantly upregulates the protein expression of VEGF and basic fibroblast growth factor, as well as the mRNA level of platelet‐derived growth factor B in myocardial tissue, increases left ventricular myocardial capillary density, and reduces myocardial infarct size, thereby exerting a pro‐angiogenic effect [195]. In the mouse model of myocardial infarction, Naoxintong capsules promote angiogenesis and reduce myocardial infarction injury by modulating the VEGF/Akt/eNOS pathway [196]. Studies have shown that in vitro in HUVECs, as well as in vivo in zebrafish and rat models, Astragaloside IV can increase the expression of eNOS and KDR and promote angiogenesis by activating the PTEN/PI3K/Akt, JAK2/STAT3, and ERK1/2 pathways [197, 198, 199]. 20(S)‐protopanaxadiol enhances angiogenesis in mice through VEGF secretion mediated by PI3K/Akt/mTOR/p70S6K and Raf/MEK/ERK signaling pathways [200]. Icariin can enhance angiogenic differentiation and inhibit autophagy in EPCs caused by oxidative stress by triggering the PI3K/Akt‐mediated mTOR/4EBP1 pathway and inhibiting the p38, MAPK/ATF2, and ERK1/2 pathways [201].

#### 5.3.4 Comprehensive Advantages of Multi‐Target Regulation

A key characteristic of TCM treatment is its comprehensive multi‐target and multi‐pathway regulatory advantages. In recent years, with the conduct of extensive in vivo and in vitro studies, significant progress has been made in understanding the role of TCM in the treatment of CMD. Existing research indicates that TCM treatment for CMD can reduce the frequency of angina attacks, alleviate damage to the microvascular endothelial barrier, inhibit the progression of myocardial reperfusion injury, enhance cardiac function, and improve long‐term prognosis. TCM‐based CMD treatment can effectively activate the NRG‐1/ErbB signaling pathway, reduce inflammation and oxidative stress, and subsequently induce VEGF secretion from endothelial cells by regulating the PI3K/Akt and Raf/MEK/ERK signaling pathways. Subsequently, coronary microcirculatory disturbances are repaired, vascular endothelial function is maintained, myocardial angiogenesis is promoted, and cardiac function is enhanced. Table 2 summarizes the evidence from TCM studies for CMD treatment based on the NRG‐1/ErbB pathway.

**TABLE 2:** Traditional Chinese medicine research on CMD based on NRG‐1/ErbB pathway.

Traditional Chinese medicine	Mechanism of action	Effect	Study type	Reference
Qiliqiangxin capsule	NRG‐1/ErbB signaling dependent on the PI3K/Akt/mTOR pathway	Improves vascular endothelial dysfunction	In vitro (CMECs), in vivo (rats)	[38, 39]
Tongxinluo capsule	NRG‐1/ErbB/AKT pathway	Inhibits endothelial‐mesenchymal transition, alleviates myocardial fibrosis	In vitro (HCMECs), in vivo (rats)	[41]
LQHTHX (Liqi Huoxue Huatan formula)	Restores NRG‐1 expression, activates NRG‐1/ErbB‐PI3K/AKT signaling	Inhibits inflammatory responses, reduces collagen deposition, improves mitochondrial function, inhibits apoptosis	In vivo (rat), in vitro (CMECs)	[36]
Shenshu Guanxin granule	Upregulates PECAM‐1/CD31 and VEGF expression	Promotes angiogenesis	In vivo (rats)	[40]
Astragalus	Induces NRG‐1 expression, activates ErbB2/4/AKT/PI3K signaling	Antiapoptotic, antioxidant, pro‐proliferative	In vitro (diabetic cardiomyopathy cell model)	[191]
Xuefu Zhuyu decoction	Upregulates VEGF expression	Promotes angiogenesis	In vivo (rats)	[193]
Qishen Yiqi dripping pill	Upregulates VEGF, bFGF, and PDGF‐B expression	Increases vascular density, reduces infarct size	Clinical, in vivo (rats)	[194, 195]
Naoxintong capsule	VEGF/Akt/eNOS pathway	Promotes angiogenesis	In vivo (mice)	[196]
Astragaloside IV	PTEN/PI3K/Akt, JAK2/STAT3, and ERK1/2 pathways	Increases eNOS and KDR expression, promotes angiogenesis	In vitro (HUVECs), in vivo (zebrafish, rats)	[197, 198, 199]
20(S)‐protopanaxadiol	PI3K/Akt/mTOR/p70S6K and Raf/MEK/ERK signaling pathways	Enhances VEGF secretion, promotes angiogenesis	In vivo (mice)	[200]
Icariin	PI3K/Akt/mTOR/4EBP1 pathway	Enhances angiogenic differentiation of endothelial progenitor cells	In vitro (endothelial progenitor cells)	[201]

## 6 Conclusions and Perspective

CMD is a significant mechanism leading to myocardial ischemia, with a high prevalence in patients with nonobstructive CAD. It is closely associated with recurrent angina attacks, heart failure, decreased quality of life, and an increased risk of major adverse cardiovascular events. This article systematically reviews the epidemiological characteristics, pathophysiological mechanisms, diagnostic assessment strategies, and treatment progress of CMD. It focuses on elucidating the multiple protective effects of the NRG‐1/ErbB pathway by regulating downstream signaling pathways such as PI3K/Akt, Raf/MEK/ERK, and RhoA/ROCK, in maintaining vascular endothelial function, inhibiting apoptosis, reducing oxidative stress, promoting myocardial angiogenesis, and regulating VR. These findings not only deepen our understanding of the pathological mechanisms of CMD but, more importantly, reveal the potential of the NRG‐1/ErbB signaling pathway as a new therapeutic target for cardiovascular diseases. Furthermore, it provides crucial theoretical support for TCM intervention in CMD through multi‐target regulation of this pathway.

TCM has shown unique advantages in treating CMD by targeting the NRG‐1/ErbB pathway, yet it still faces substantial challenges. Existing research suggests that various TCM compound formulas and active ingredients can play a positive role in improving vascular endothelial function, inhibiting inflammation and oxidative stress, promoting angiogenesis, and reducing myocardial damage by regulating NRG‐1/ErbB and its related signaling networks. However, high‐quality clinical studies (such as multicenter, large‐sample, randomized controlled trials [RCTs]) are relatively lacking, with most evidence coming from small‐sample observational studies, limiting the reliability and generalizability of findings. Although some studies have attempted to predict potential targets using modern technologies such as network pharmacology and molecular docking [202], these have not yet been validated through in vitro cell experiments or in vivo animal models, making precise application difficult. Therefore, future research should focus on strengthening high‐quality clinical studies of TCM in CMD treatment and clarifying its mechanisms of action using modern pharmacological techniques, thereby promoting the standardization of integrated TCM and Western medicine treatment.

Future research can be further advanced in the following aspects: (1) by combining single‐cell sequencing, spatial transcriptomics, multi‐omics integration, and gene editing technologies, focusing on different pathophysiological subtypes of CMD to analyze the dynamic regulatory mechanisms in depth of the NRG‐1/ErbB pathway in endothelial cells, smooth muscle cells, cardiomyocytes, and inflammatory cells; (2) strengthening mechanism validation research, using techniques such as gene knockout, receptor blocking, specific agonists/inhibitors, and chemical probes to clarify the direct action modes and key targets of active ingredients of TCM on the NRG‐1/ErbB pathway; (3) to construct a more clinically relevant CMD animal model and translational research system, and to systematically analyze the in vivo pharmacokinetic behavior of active ingredients of TCM by combining modern drug analysis technology, clarify their oral bioavailability and distribution characteristics in coronary microvascular target organs, and improve the consistency between basic research and clinical disease phenotypes; (4) conducting large‐sample, high‐quality, prospective clinical trials to systematically evaluate the efficacy, safety, and long‐term benefits of TCM and related emerging therapies for CMD; and (5) promoting the integrated development of TCM and Western medicine with precision medicine, and exploring synergistic intervention models of TCM and conventional drugs in different CMD subtypes.

In summary, CMD is a typical example of a series of structural and functional abnormalities affecting the coronary microcirculation. Elucidating its pathological mechanisms and developing precision treatment strategies are important directions for future research and clinical practice. The NRG‐1/ErbB signaling pathway, as a crucial link connecting endothelial protection, anti‐oxidative stress, anti‐remodeling, and pro‐angiogenesis, provides a new entry point for CMD mechanism research and treatment innovation. With the continuous accumulation of basic research, translational medicine, and high‐quality clinical evidence, TCM and modern medicine are expected to achieve deeper integration in the prevention and treatment of CMD, thereby providing a more solid theoretical foundation for improving the diagnosis and treatment level and long‐term prognosis of CMD patients.

## Author Contributions

Z.G. conceptualized and visualized the study, and wrote the original draft. M.Z.Z. contributed to writing, review and editing, and visualization. X.J.N. and Y.J.D. wrote the original draft and contributed to visualization. L.S.S. and X.D.W. conceptualized the study and contributed to writing, review and editing. J.Y.Z. and T.Z.L. conceptualized and visualized the study. X.L.W. conceptualized, supervised, acquired funding for the study, and contributed to writing, review and editing. All authors have read and approved the final manuscript.

## Funding

This work was supported by the National Natural Science Foundation of China (No. 82174326); the Innovation Team and Talents Cultivation Program of National Administration of Traditional Chinese Medicine (No. ZYYCXTD‐C‐202203); the Innovation Team Development Plan of the Ministry of Education—Research on the Prevention and Treatment of Cardiovascular Diseases in Traditional Chinese Medicine (No. IRT_16R54); the Tianjin Science and Technology Planning Project (23JCZXJC00060); the Beijing‐Tianjin‐Hebei Basic Research Cooperation Project (J230037); the Tuoxin Project Fund Research Project of the First Teaching Hospital of Tianjin University of Traditional Chinese Medicine (TX2025027); and the Scientific Research Program of Tianjin Municipal Education Commission (Natural Science) (2025KJ112).

## Ethics Statement

The authors have nothing to report.

## Conflicts of Interest

The authors declare no conflicts of interest.

## Data Availability

The authors have nothing to report.
